# The Prevention of Rabies

**Published:** 1898-01-08

**Authors:** 


### MEDICAL PROGRESS AND HOSPITAL CLINICS.

[*The Editor will be glad to receive offers of co-operation and contributions from members of the profession. All letters should be addressed to* THE EDITOR, AT THE OFFICE, 28 & 29, SOUTHAMPTON STREET, STRAND, LONDON, W.C.]

### THE PREVENTION OF RABIES.

In an article which appeared in THE HOSPITAL on December 18th, we showed how useless for the prevention of rabies was the imposition of local muzzling orders by local authorities. Although the hearty co-operation of all local authorities is necessary if rabies is to be abolished, it is abundantly clear that if this co-operation is to be of any real service the organisation, the determination where and when muzzling is to be enforced, must lie with a central body. The absurdity of attempting to obtain control over so widely spread a disease as rabies by the isolated and independent action of local bodies may be understood when we mention that Lancashire alone is governed in this matter by no less than 34 separate authorities, many of which are boroughs lying like islands in the midst of rural areas. In Ireland the matter is even worse, for in that country there are no less than 880 local authorities possessing power to impose muzzling regulations, the majority of them exercising jurisdiction over too restricted an area to permit the use of their powers to any good purpose.

If, then, muzzling pure and simple were our only resource for the suppression of rabies, the conclusion would be irresistible that it should be made universal. In the words of the report of a departmental committee on the subject, local muzzling "produces the maximum of local irritation with the minimum of general and permanent good."

On the other hand, as against the attempt to enforce universal muzzling, it is maintained by many who have studied the matter that, even in face of the most stringent regulations with that object, a good deal of laxity is apt to creep in, and that if this happens in districts where the presence of rabies is recognised and the danger of hydrophobia is acknowledged, it is altogether improbable that the muzzling would be uniformly and thoroughly carried out in other parts of the country. It becomes, then, a matter of considerable interest to inquire what other means we possess of dealing with this very serious disease, and to ascertain how far such means as have been employed with this object have been successful.

The first thing to note is the very different extent to which the disease prevails in different parts of the country. The three districts which are the main hotbeds of rabies are Lancashire and the West Riding of Yorkshire, the area of which Birmingham is the centre, and London. It is to be noted that all these are largely urban districts; and this is of some importance because the teachings of experience seem to show that under the influence of measures other than muzzling, rabies in the country tends to die out, while in towns such measures without muzzling have but small effect. What we wish to draw attention to is the fact that muzzling as used by the Board of Agriculture is but part of a large scheme for the suppression of rabies which is in constant operation all the country over, and that by the judicious use of muzzling orders over certain large areas as occasion may demand, and always as part of such a general scheme, there is much hope of completely stamping out the disease. In fact, according to those entrusted with the work, there is more hope of good result from what is now being done than there would be from any general muzzling, in consequence of the difficulty of enforcing such muzzling in certain parts of the country.

Rabies is but one of a series of highly infectious diseases with which the Board of Agriculture has to deal, and for dealing with which it is in possession of a complete machinery extending all over the country.

Notification, inspection, and the immediate destruction of all animals which have been exposed to infection are the measures on which trust is mainly placed. These have been effectual in suppressing cattle-plague, foot-and-mouth disease, and pleuro-pneumonia—diseases the infectivity of which is far more difficult to control than the exclusively "bite infection" of rabies; while against rabies, in regard to which there is an additional difficulty arising from the tendency the affected dogs have to wander, there is the additional means of control given by muzzling. Every case is at once reported at Whitehall. If there is any doubt about it a complete examination is made and full tests are applied by experts. In every case the whole history of the affected dog is followed up, it may be through the districts of half a dozen local authorities, and every dog known to have run risk of infection is destroyed. Specially skilled inspectors, accustomed to make such inquiries, are employed on this errand, and the greatest pains are taken to trace out every dog known to have been touched. In the country this of itself is often enough to prevent further mischief, for people in the country know each other and each other's dogs. But in towns this sort of inquiry can hardly ever be sufficient, and a muzzling order is the only resource.

A rabid dog was found and killed at Tonbridge, where it had bitten several others. It was traced back to Croydon, whence it had wandered. Upwards of forty dogs which were found to have been exposed to infection by that one dog in its wanderings through the different districts were all destroyed, and the outbreak came to an end. This sort of thing is going on all over the country, and it is maintained by those who have the management of it that this notification, inspection, and tracing out of every case is more effectual than an unpopular and therefore half-hearted universal muzzling. Nevertheless, the efficacy of the muzzle when properly enforced by a central authority is made very plain by the history of the past few years, as also is its uselessness when in the hands of local bodies.

During the four years 1889 to 1892 muzzling was imposed by a central authority over wide areas, whilst in the period from 1893 to the beginning of 1896 it was practically left to the local authorities. The following are the figures for these years:—

| | | | | | |
|---|---|---|---|---|---|
| Central Control | In 1889 | there were | 312 | cases. |
| | ,, 1890 | ,, | ,, | 129 | ,, |
| | ,, 1891 | ,, | ,, | 79 | ,, |
| | ,, 1892 | ,, | ,, | 38 | ,, |
| Local Control | In 1893 | there were | 93 | cases. |
| | ,, 1894 | ,, | ,, | 248 | ,, |
| | ,, 1895 | ,, | ,, | 672 | ,, |

In London the effect of muzzling is shown by the following table:—

| | | | | | |
|---|---|---|---|---|---|
| Muzzling | In 1889 | there | were | 123 | cases |
| | ,, 1890 | ,, | ,, | 32 | ,, |
| | ,, 1891 | ,, | ,, | 13 | ,, |
| | ,, 1892 | ,, | ,, | 3 | ,, |
| No Muzzling | In 1893 | there | were | 8 | cases. |
| | ,, 1894 | ,, | ,, | 12 | ,, |
| | ,, 1895 | ,, | ,, | 46 | ,, |

Such then being the means at the disposal of the Board of Agriculture for the suppression of rabies, it remains for us to see how far they have proved effectual, and what hope there is that by their aid the disease may be stamped out.

Mr. Long has kindly furnished us with a map of England and Wales on which the areas under muzzling orders are distinguished and every case of rabies, from January 1st to December 31st, 1897, is marked. An examination of this map shows that during the year 145 cases occurred, and yet that, although less than a third of the whole country is now muzzled, only ten of the above cases occurred outside the area now under orders. It is interesting to observe that only four of these cases were discovered at any distance from the muzzled areas; that, of these, two occurred so far back as last January and one in February, and that one, which took place in Essex in November, is still looked upon as a doubtful case. The other six cases were all located on the fringe of the area now muzzled. Other maps which have been shown to us at the offices of the Board of Agriculture illustrate the thoroughness with which muzzling has been made to follow the outbreaks of the disease, so that if we bear in mind that in regard to some of the few outbreaks which have not been followed by orders the dog has been traced, and the authorities have been able to satisfy themselves that all dogs which had been exposed to infection had been destroyed, we see that the area of the muzzling does now practically cover the area of the rabies.

A careful examination of the dates also shows how steadily the disease has already diminished. In the quarter ending June 30th there were 41 cases, in that ending September 30th there were 39, and in that ending December 31st there were 20. In a letter, dated December 31st, we are informed that, with the exception of a case in Devonshire, there has been no case outside the muzzling order which surrounds London since November 1st, a case which occurred in Essex on November 27th being still one of doubt.

What the Board is endeavouring to do is to stamp out the disease, not by muzzling alone, but by all the statutory powers at its disposal—by notification, inspection, destruction of infected dogs, the application of muzzling orders over large areas as may be found necessary from time to time, and by the regulation and control of imported dogs.

As things stand at present they certainly show a good record, and there seems much hope that they may be able to attain their object. They also desire, as a permanent arrangement, that the local authorities should be empowered to make regulations enforcing the wearing of collars, and other measures for diminishing the evils which arise from the number of stray and ownerless dogs, for dogs are responsible for much mischief besides rabies. But in regard to the suppression of that disease, they believe that concerted measures, such as we have described as being in operation at the present time, are essential, and that they will be sufficient to stamp out rabies and prevent it from being introduced afresh. Nevertheless, it is clear, and we believe that this opinion is also held by the officials, that if the simple imposition of muzzling alone had to be trusted to, that muzzling would have to be universal and long-continued. What they are doing now, however, seems to Mr. Long and those working with him to be the better plan.

